# TNF-α-induced Tim-3 expression marks the dysfunction of infiltrating natural killer cells in human esophageal cancer

**DOI:** 10.1186/s12967-019-1917-0

**Published:** 2019-05-20

**Authors:** Yujia Zheng, Yu Li, Jingyao Lian, Huiyun Yang, Feng Li, Song Zhao, Yu Qi, Yi Zhang, Lan Huang

**Affiliations:** 1 Biotherapy Center, The First Affiliated Hospital, Zhengzhou University, Building #9, 1 Jianshe East Road, Zhengzhou, 450052 Henan China; 2 Department of Thoracic Surgery, The First Affiliated Hospital, Zhengzhou University, Zhengzhou, 450052 Henan China

**Keywords:** Tumor microenvironment, NK cells, Tim-3, TNF-α, Esophageal cancer

## Abstract

**Background:**

Impairment of natural killer (NK) cell activity is an important mechanism of tumor immunoevasion. T cell immunoglobulin domain and mucin domain-3 (Tim-3) is an activation-induced inhibitory molecule, inducing effector lymphocyte exhaustion in chronic viral infection and cancers. However, its function in NK cells in human esophageal cancer remains unclear.

**Methods:**

We prospectively collected peripheral blood and tumor samples from 53 patients with esophageal cancer. Peripheral and tumor-infiltrating NK cells were analyzed for Tim-3, Annexin V, CD69, CD107a and IFN-γ expression by flow cytometry. Quantitative real-time PCR was used to test relative mRNA expression of IFN-γ, granzyme B, perforin and NKG2D in sorted Tim-3^+^ NK cells and Tim-3^−^ NK cells, respectively. NK cells isolated from healthy donors were treated with recombinant TNF-α to induce Tim-3 expression. Tim-3 and TNF-α mRNA levels in tumor tissues were measured in both humans and mice. Finally, associations between NK cell frequencies with pathological parameters were investigated.

**Results:**

We observed up-regulation of Tim-3 expression on NK cells from esophageal cancer patients, especially at the tumor site. Furthermore, tumor-infiltrating NK cells with high Tim-3 expression exhibited a phenotype with enhanced dysfunction. In vitro, Tim-3 expression on NK cells isolated from blood of healthy donors can be induced by recombinant TNF-α via NF-κB pathway. In both animal models and patients, the Tim-3 level was positively correlated with TNF-α expression in esophageal cancer tissues. Finally, higher Tim-3 level on tumor-infiltrating NK cells is correlated with tumor invasion, nodal status and poor stage in patients with esophageal cancer.

**Conclusions:**

Taken together, Tim-3 may play a crucial role to induce NK cell dysfunction in tumor microenvironment and could serve as a potential biomarker for prognosis of esophageal cancer.

**Electronic supplementary material:**

The online version of this article (10.1186/s12967-019-1917-0) contains supplementary material, which is available to authorized users.

## Background

Esophageal cancer is one of the leading causes of cancer-related death worldwide. Globally, approximately half of all cases occur in China [1]. Despite of great advancements in early detection, precision diagnosis and combination therapy, the overall 5-year survival rate of esophageal cancer is still unsatisfactory [2]. Evasion of immune surveillance is an important hallmark of cancer, acquired during tumor initiation and development. Dysfunction or exhaustion of T lymphocytes in tumor microenvironment has been recognized as a key mechanism to the pathogenesis of human malignant diseases [3]. Notably, Immunotherapy aimed at restoring anti-tumor activity of T lymphocytes has become a pillar of cancer therapy [4].

Natural killer (NK) cells are the main cells that constitute innate immunity and play an important role in the anti-tumor immune surveillance [5]. Many studies have shown that the number of infiltrating NK cells in tumor tissues is significantly related to the prognosis of cancer patients, including esophageal cancer [6, 7]. NK cells within tumor microenvironment are often impaired by many different mechanisms, such as reduced numbers, imbalances between activating and inhibitory receptors, and immunosuppressive cytokines [8]. Recently, dysfunctional NK cells are characterized by surface expression of co-inhibitory receptors [9]. It has been reported that programmed cell death protein 1 (PD-1) on NK cells indicates poor survival of esophageal cancer and blockade of PD-1 signaling restores NK cell function [7, 10].

Besides PD-1, T-cell immunoglobulin domain and mucin domain-3 (Tim-3) is another potential exhaustion marker induced by chronic infections or cancers. Tim-3 was first discovered on Th1 cells and exhibited functions as a co-inhibitory receptor that down-regulates the activity of tumor infiltrating lymphocytes (TIL) in different types of cancer [11–13]. Blockade of Tim-3 signaling restores TIL functions in vitro and in vivo [14]. Later, Tim-3 has also been found on the surface of innate immune cells, including dendritic cells, macrophages, and NK cells [15]. Importantly, high Tim-3 expression on innate immune cells may mediate suppressive responses [16]. Early research suggests that Tim-3 functions as an inducible receptor on human NK cells to enhance IFN-γ production in response to galectin-9 [17]. However, later studies have shown that Tim-3^+^ NK cells from cancer patients produce lower levels of IFN-γ and are functionally exhausted [12]. Recent studies reported that a high percentage of Tim-3^+^ NK cells was associated with poor prognosis in patients with gastric cancer and lung adenocarcinoma [18, 19]. Furthermore, Tim-3 blockade can increase the antitumor activity of NK cells from melanoma patients [20]. However, the relationship between Tim-3 expression on NK cells and human esophageal carcinoma is not well understood.

In this study, we characterized the phenotypes and functions of NK cells from esophageal carcinoma in human and mice. We found that Tim3^+^ NK cells were functionally defective and correlated with poor prognosis in esophageal cancer patients. Mechanistically, Tim-3 was induced by tumor necrosis factor-α (TNF-α) through NF-κB signaling pathway. These findings indicate Tim-3 as a potential prognostic marker and a promising therapeutic target in esophageal cancer.

## Materials and methods

### Esophageal cancer patients

Blood and tissue samples were collected from 52 patients with untreated esophageal cancer in the First Affiliated Hospital of Zhengzhou University between September 2016 and April 2018. The demographic information of patients was summarized according to their age, gender, tumor site and pathological stage (Additional file 1: Table S1). Blood samples from healthy donors were obtained from the local blood bank.

### Isolation of lymphocytes

Peripheral blood mononuclear cells (PBMC) were isolated by Ficoll-Hypaque density gradient centrifugation. The Tumor Dissociation Kit and the gentleMACS Dissociator (Miltenyi Biotec) were used for TIL isolation from tumor tissues according to the manufacturer’s instructions. Cell suspensions were then isolated by Ficoll-Hypaque to remove red cells and dead cells. The fresh TIL were immediately used for flow cytometry analysis or sorting.

NK cells were purified from PBMC of healthy donors and esophageal cancer patients using MACS NK kit (Miltenyi Biotec). Tim-3^+^ NK cells and Tim-3^−^ NK cells were sorted from TIL by MoFlo XDP cytometer (Beckman Coulter) and then used for gene expression analysis from small number of cells.

### Flow cytometry

For surface phenotype assessment, cells were stained with fluorochrome-conjugated primary antibodies for 30 min at 4 °C. An isotype control was performed for each sample. For cytokine detection, cells were stimulated with PMA and ionomycin (Sigma) in the presence of a protein transport inhibitor brefeldin A (BioLegend) for 6 h, and then stained with surface fluorochrome-conjugated primary antibodies. After fixing with 4% paraformaldehyde and permeabilizing with 0.1% saponin, cells were finally stained intracellularly with specific antibodies against human cytokines. Samples were stained with 7-AAD viability staining solution (BioLegend) to exclude dead cells and analyzed with a FACSCanto II cytometer (BD Biosciences). Details of the antibodies were summarized in Additional file 1: Table S2.

### RNA extraction and quantitative PCR

Total RNA was extracted from tissues using RNAiso Plus (TakaRa) according to the manufacturer’s instructions. Total RNA was quantified by a NanoDrop spectrophotometer (Thermo Fisher Scientific), and cDNA was synthesized by using PrimeScript RT reagent Kit with gDNA Eraser (TakaRa). Quantitative PCR (qPCR) reactions using specific primers and SYBR Green qPCR Master Mix (Roche) were performed in an Mx3005P qPCR System (Agilent Technologies). For detection of small cell samples, a one-step qPCR reaction was performed using CellsDirect One-Step qRT-PCR kit (Invitrogen). The sequences of PCR primers was listed in Additional file 1: Table S3.

### TNF-α treatment of human NK cells in vitro

Purified human NK cells were suspended in modified GT-T551 medium (TaKaRa) supplemented with 10% FBS (HyClone), 200 IU/mL recombinant human IL-2, 100 units/mL penicillin and 100 μg/mL streptomycin (Sigma). NK cells were treated with recombinant human TNF-α (Peprotech), NF-κB inhibitor (Selleck) or TNF-α inhibitor (Shanghai Saijin) for 48 h, respectively. Surface Tim-3 expression on NK cells was then evaluated by flow cytometry.

### Murine esophageal carcinoma model

Male C57BL/6 mice (7-week-old) were purchased from Beijing Vital River Laboratory Animal Technology and randomly divided into two groups: control (n = 10) and 4-NQO (n = 18). The carcinogen 4-NQO (Sigma) was dissolved in propylene glycol (Sigma) at 5 mg/mL and diluted in the drinking water to 100 µg/mL. After 12 weeks of 4-NQO treatment, mice were followed by sterile distilled water for another 16 weeks before sacrifice. Esophageal tissues were used for histopathology and qPCR analysis, respectively. Spleens were obtained from each animal for lymphocyte analysis by flow cytometry.

### Statistical analysis

All statistical analyses were performed by using the Graph-Pad Prism 7 software. The significance of variation between groups was evaluated using Student’s t-test. Data are presented as the mean ± SEM from at least three independent experiments. Differences were considered to be statistically significant when *p *< 0.05 (*p < 0.05; **p < 0.01; ***p < 0.001).

## Results

### Tim-3 is up-regulated in peripheral and tumor infiltrating NK cells from patients with esophageal cancer

**Fig. 1:**
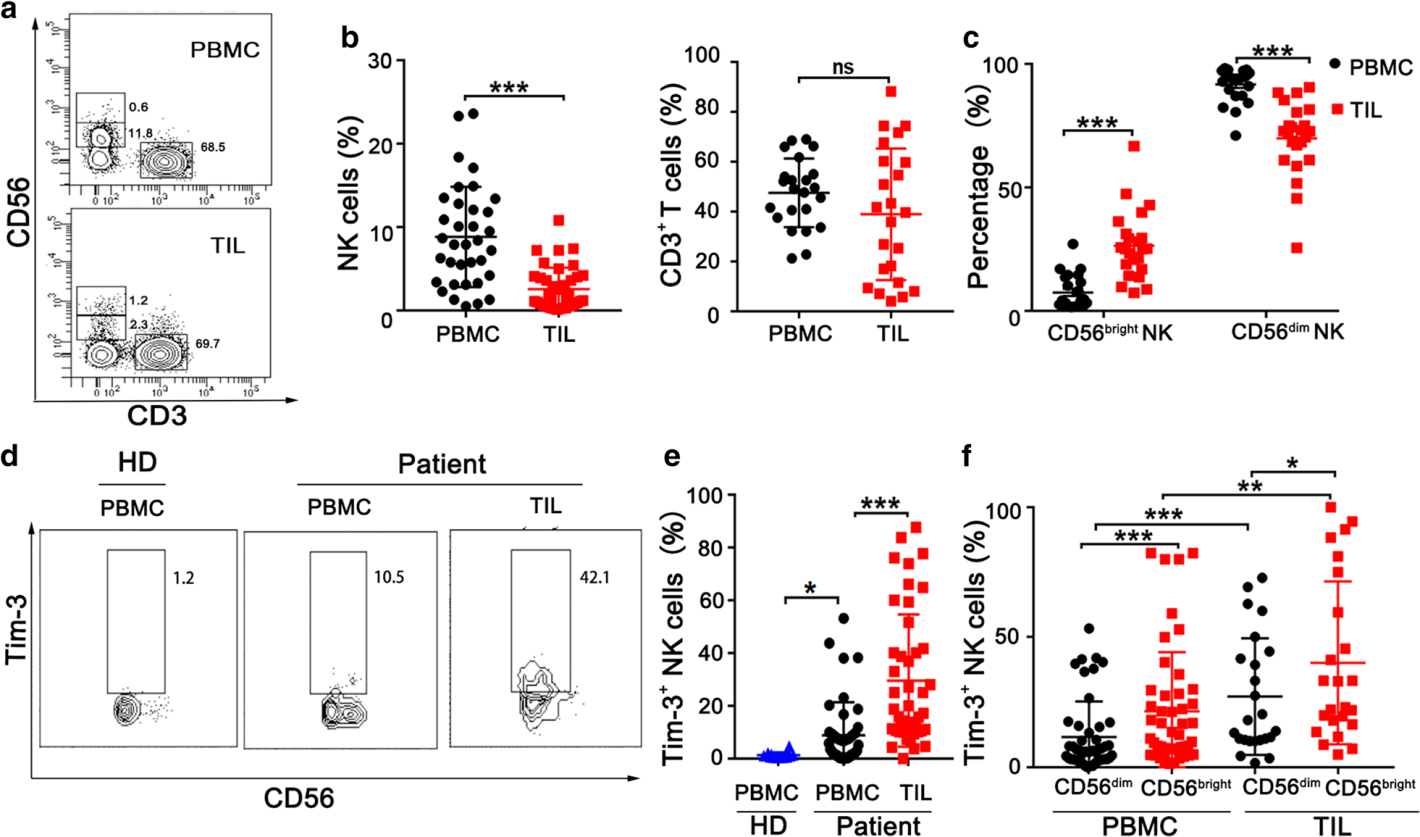
Increased Tim-3 expression on circulating and intratumoral NK cells from esophageal cancer patients. **a** Representative flow plots of NK (CD3^−^CD56^+^) cells and T (CD3^+^CD56^−^) cells in paired PBMC and TIL from an esophageal cancer patient. **b** Proportions of NK cells and CD3^+^ T cells in paired PBMC and TIL from esophageal cancer patients. **c** Proportions of CD56^bright^ and CD56^dim^ NK cell subsets in paired PBMC and TIL in patients with esophageal cancer. **d** Representative flow plots of Tim-3^+^ NK cells in a esophageal cancer patient and a healthy donor (HD). **e** Proportions of Tim-3^+^ NK cells in patients with esophageal cancer (n = 44) and healthy donors (n = 14). **f** Proportions of Tim-3^+^ on CD56^dim^ and CD56^bright^ NK cell subsets in paired PBMC and TIL in patients with esophageal cancer. *p < 0.05; **p < 0.01; ***p < 0.001; ns, not significant

Many studies have shown that effector lymphocytes are exhausted in tumor microenvironment, but the functions of NK cells in esophageal cancer are still obscure. Using flow cytometer, we first examined lymphocyte subsets in PBMC and TIL from esophageal cancer patients (Fig. 1a). Notably, NK cells were dramatically decreased in tumor tissues. However, there was no statistic difference in the frequency of T cells (Fig. 1b). Traditionally, NK cells can be divided to two major subsets based on CD56 expression: CD56^bright^ and CD56^dim^ [21, 22]. CD3^−^CD56^dim^ NK cells have function of cytotoxic and mediate antibody-dependent cellular cytotoxicity (ADCC). In contrast, CD3^−^CD56^bright^ subset produces abundant cytokines but shows weakly cytotoxic features before activation. Next, we analyzed the two subsets of NK cells and found that the frequency of CD56^dim^ NK cells significantly reduced in TIL compared with PBMC (Fig. 1c).

Tim-3 is a potential co-inhibitory molecule expressed on T cells in the context of progressive cancers. In this study, we examined Tim-3 expression in peripheral and tumor-infiltrating NK cells from esophageal cancer patients (Fig. 1d). Compared with NK cells from healthy donors, peripheral NK cells from esophageal cancer patients expressed significantly higher levels of surface Tim-3. Moreover, Tim-3 expression further increased in tumor-infiltrating NK cells (Fig. 1e) and T cells (data not shown). We also detected several other co-inhibitory molecules (PD-1, TIGIT, and LAG-3) on the surface of NK cells in 5 esophageal cancer patients. Compared with peripheral NK cells, tumor-infiltrating NK cells displayed an increase of LAG-3 expression but no significant difference in the context of PD-1 and TIGIT expression (Additional file 1: Figure S1). We further analyzed Tim-3 expression in two NK subsets and found that CD56^bright^ NK cells expressed higher levels of Tim-3 than CD56^dim^ NK cells in both PBMC and TIL (Fig. 1f). These results clearly demonstrate that NK cells in human esophageal cancer tissues display a remarkable decrease of CD56^dim^ subset but a significant increase of Tim-3 expression.

### Tim-3 is associated with an impairment of NK cell functions

**Fig. 2:**
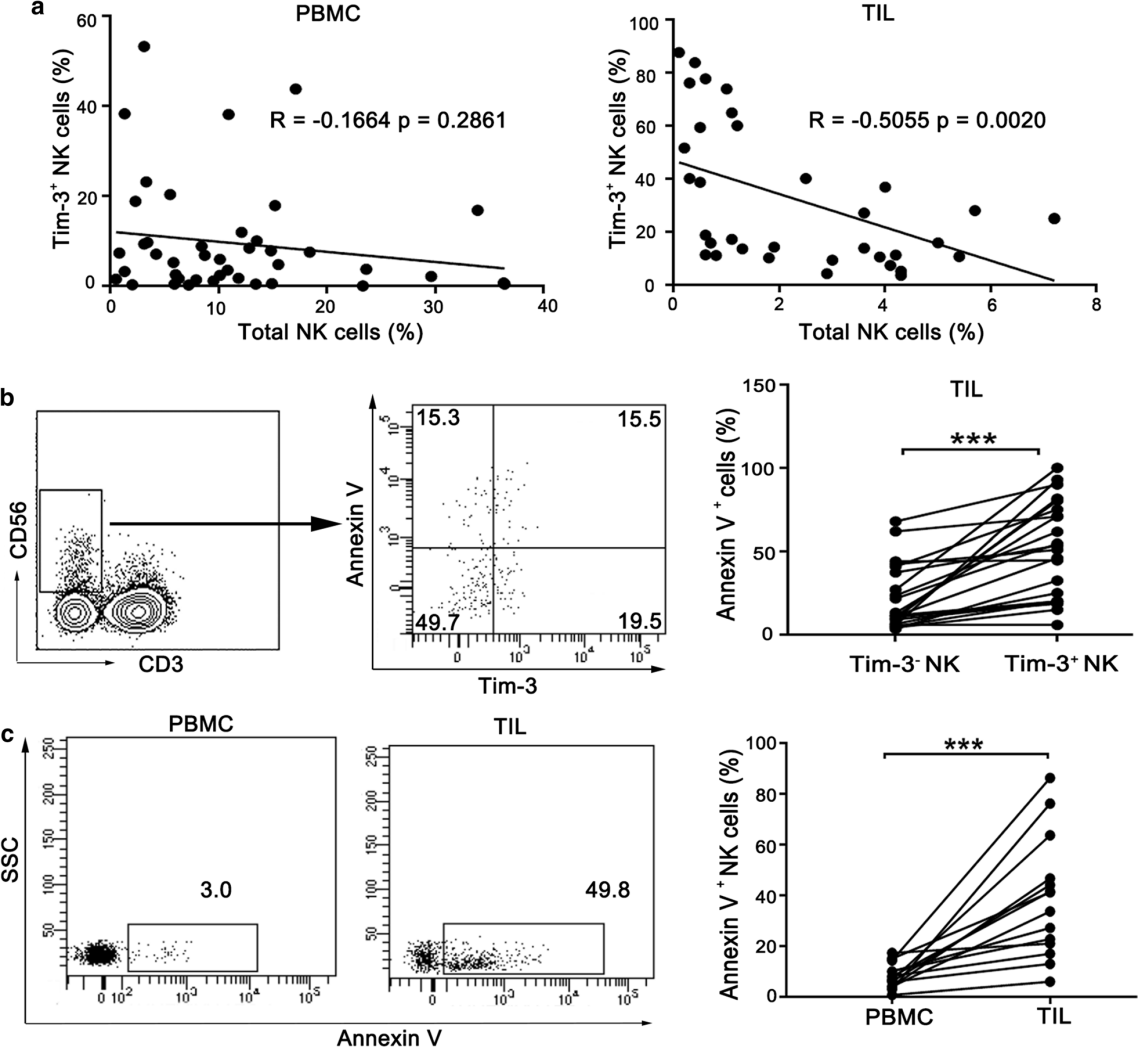
Tim-3 expression is associated with apoptosis of tumor-infiltrating NK cells. **a** Correlation analysis between Tim-3^+^ NK cells and total NK cells in PBMC and TIL from esophageal cancer patients. **b** TIL were stained with antibodies to CD3, CD56, Tim-3 and Annexin V. The flow data are representative of 23 patients with esophageal cancer (left panel) and the results are shown as bar graphs (right panel). **c** NK cells in PBMC and TIL were examined for Annexin V binding, respectively. The flow data are representative of 14 patients with esophageal cancer (left panel) and the results are shown as bar graphs (right panel). ***p < 0.001; ns, not significant

The role of Tim-3 in NK cells remains elusive under the microenvironment of human esophageal cancer. Interestingly, we found a significant negative correlation between Tim-3 expression and cell frequency in tumor-infiltrating NK cells but not peripheral NK cells from esophageal cancer patients (Fig. 2a). Therefore, we hypothesized that the decreased frequency of NK cells in tumor site may be induced by Tim-3 expression on NK cells, through promotion of NK cell death. To this end, we analyzed apoptosis of tumor-infiltrating NK cells by Annexin V staining. In tumor-infiltrating lymphocytes, Tim-3^+^ NK cells exhibited a greater tendency toward apoptosis compared with the Tim-3^−^ NK cells (Fig. 2b). In addition, tumor-infiltrating NK cells are more susceptible to apoptosis compared with peripheral NK cells (Fig. 2c). Increased proportion of apoptotic cells is a sign of lymphocyte exhaustion. We speculate that up-regulation of Tim-3 is associated with NK cell dysfunction in esophageal cancer microenvironment.

**Fig. 3:**
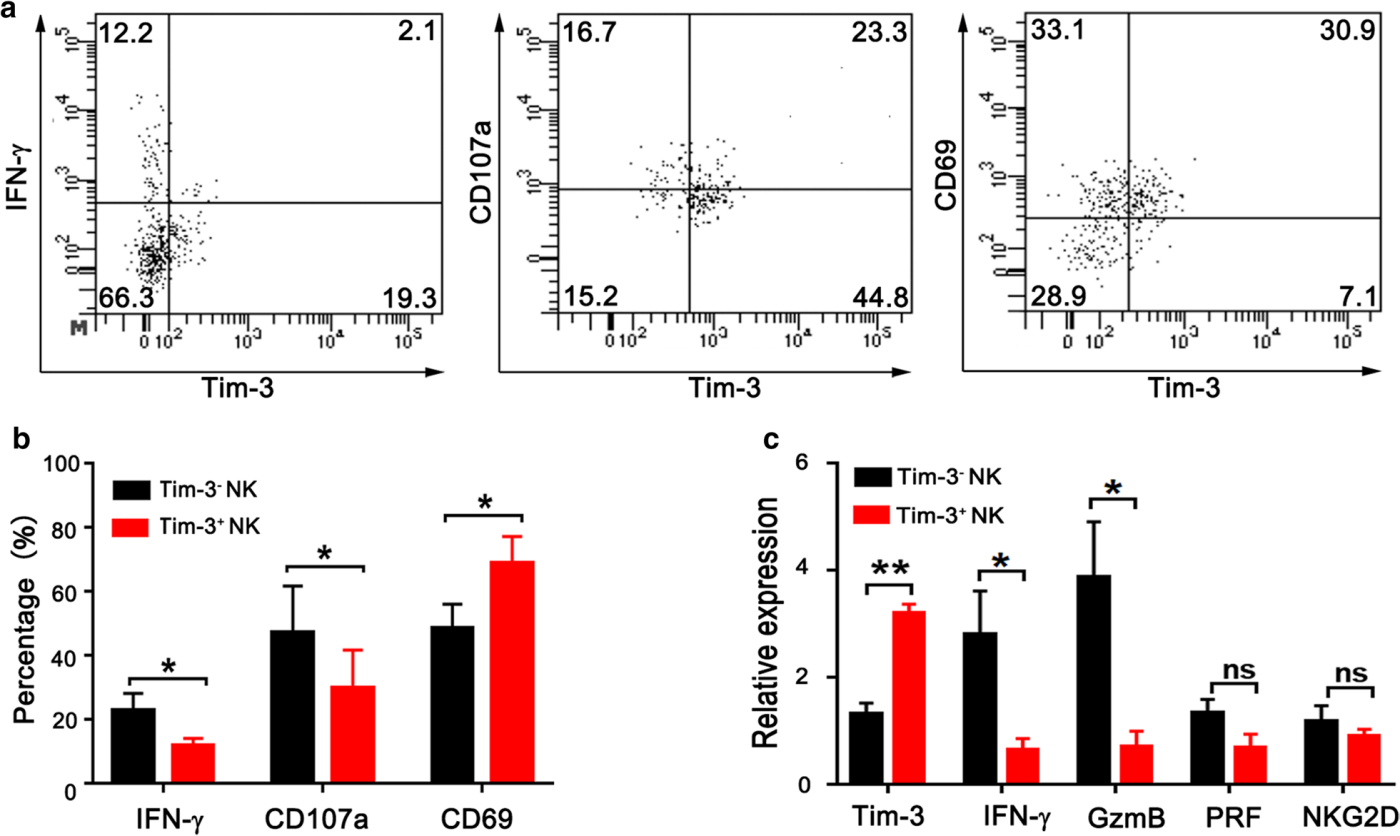
Tim-3^+^ NK cells display weaker activities than Tim-3^−^ NK cells in TIL from esophageal cancer patients. **a** Representative flow plots of IFN-γ, CD107a and CD69 expression on Tim-3^+^ NK cells and Tim-3^−^ NK cells. **b** Proportions of IFN-γ^+^ cells (n = 7), CD107a^+^ cells (n = 5) and CD69^+^ cells (n = 8) in Tim-3^+^ NK cells and Tim-3^−^ NK cells, respectively. **c** Relative mRNA expression of Tim-3, IFN-γ, granzyme B (GzmB), perforin (PRF) and NKG2D in sorted Tim-3^+^ NK cells and Tim-3^−^ NK cells, respectively (n = 5). *p < 0.05; **p < 0.01; ns, not significant

To further characterize Tim-3^+^ NK cells, we examined IFN-γ production, cytotoxic activity and activation marker of NK cell subsets from human esophageal tumor tissues (Fig. 3a). In comparison with Tim-3^−^ NK cells, Tim-3^+^ NK cells produced less IFN-γ under stimulation with PMA and inomycin. In addition, they exhibited a significant decrease of cytotoxicity as measured by CD107a expression. Interestingly, Tim-3^+^ NK cells expressed higher levels of CD69 when compared with Tim-3^−^ NK cells (Fig. 3b). CD69 is an early activation marker of lymphocytes. In tumor microenvironment, CD69 expression may represent over-activation of lymphocytes [23, 24]. Finally, we sorted tumor-infiltrating NK cells into Tim-3^+^ NK cells and Tim-3^−^ NK cells, and examined mRNA levels of key molecules related to NK cell activity by qPCR. Consistently, lower levels of IFN-γ and granzyme B were detected in Tim-3^+^ NK cells (Fig. 3c). These results strongly suggest that Tim-3^+^ NK cells following early activation were functionally impaired as shown by defects in cytokine production and cytotoxicity.

### TNF-α induces the expression of Tim-3 in NK cells through the NF-κB Signaling Pathway

**Fig. 4:**
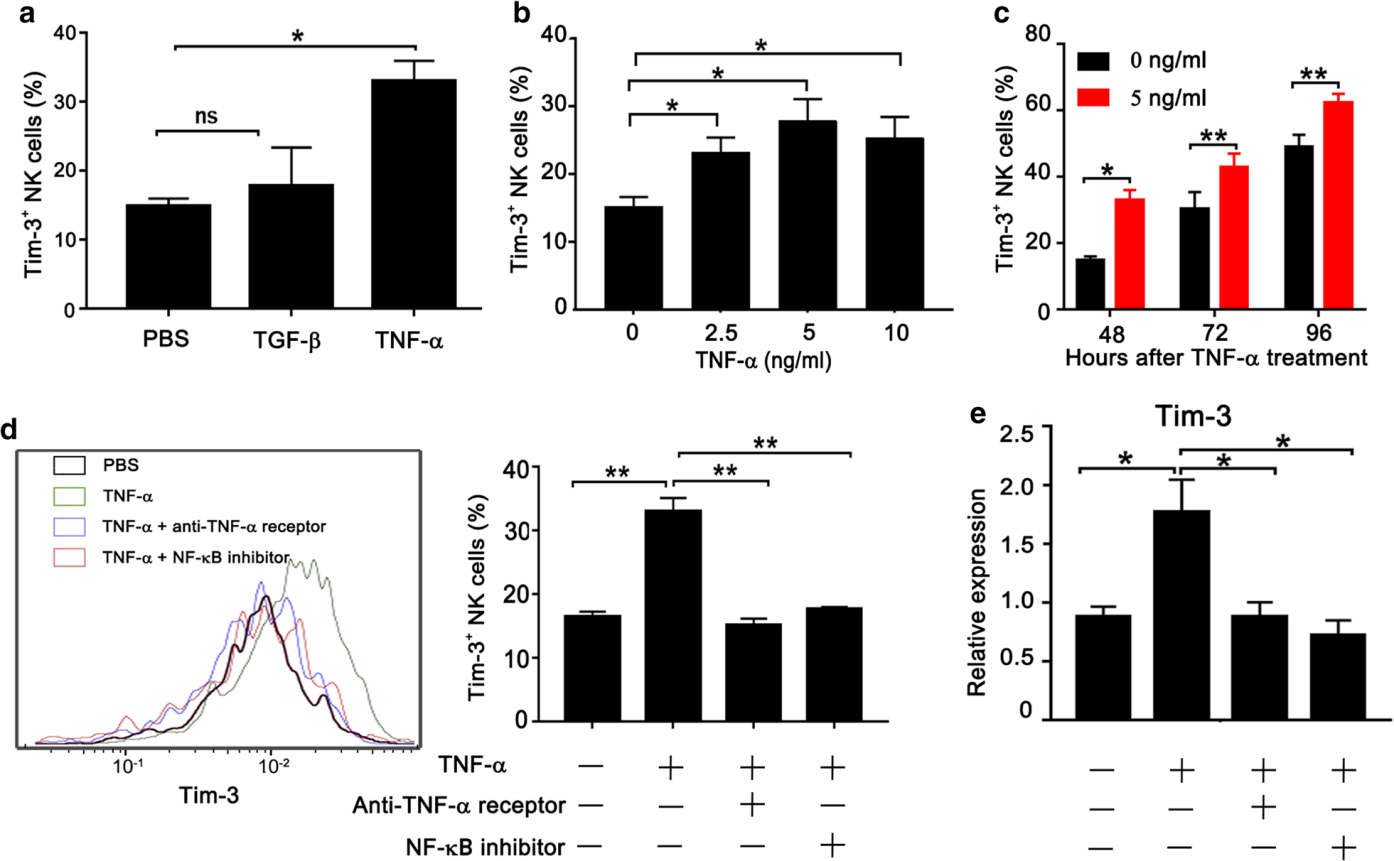
TNF-α induces expression of Tim-3 on human NK cells in vitro. **a** Peripheral NK cells purified from healthy donors were treated with recombinant human TGF-β (10 ng/mL) or TNF-α (5 ng/mL) in the presence of IL-2 for 48 h. The surface expression of Tim-3 was measured by flow cytometry. **b** Human NK cells were stimulated with different concentrations of TNF-α for 48 h before flow cytometry analysis. **c** Human NK cells were stimulated with 5 ng/mL TNF-α for the indicated time before flow cytometry analysis. NK cells were treated with TNF-α inhibitor or NF-κB inhibitor in the presence of 5 ng/mL TNF-α for 48 h. Tim-3 protein (**d**) or mRNA (**e**) was measured by flow cytometry or qPCR analysis, respectively. *p < 0.05; **p < 0.01; ns, not significant

TNF-α and TGF-β are important inflammatory factors in the tumor microenvironment [25, 26]. It has been reported that TNF-α can induce Tim-3 expression on T cells but Tim-3 expression on macrophages can be boosted by TGF-β [27, 28]. However, the mechanism of Tim-3 overexpression on NK cells is not clear, especially in tumor microenvironment. We assumed that TNF-α or TGF-β could also induce Tim-3 expression on NK cells. To this purpose, we cultured purified NK cells form healthy donors with recombinant human TNF-α or TGF-β protein respectively. As shown in Fig. 4a, TNF-α but not TGF-β could induce surface Tim-3 expression on NK cells. Moreover, we confirm that TNF-α leads to an increase of Tim-3 expression on NK cells in dose-dependent (Fig. 4b) and time-dependent manners (Fig. 4c). To maintain the activity of NK cells, a low concentration of IL-2 was added to the culture system. As previously reported [20], we also observed that IL-2 alone induced expression of Tim-3 on NK cells in the absence of TNF-a (Fig. 4c). To validate the role of TNF-α in Tim-3 expression on NK cells, we added TNF-α inhibitor to NK cell culture. As expected, TNF-α inhibitor completely blocked Tim-3 expression induced by TNF-α (Fig. 4d). NF-κB is a well-known transcription factor that is activated by TNF-α. Therefore, we hypothesized that NF-κB mediated TNF-α-induced Tim-3 expression on NK cells. To test this hypothesis, we added a NF-κB inhibitor into NK cells cultured with TNF-α and found that TNF-α-induced Tim-3 expression dramatically decreased in the presence of NF-κB inhibitor (Fig. 4d). Finally, we examined Tim-3 mRNA expression in human NK cells under stimulation with TNF-α. Consistently, TNF-α failed to induce Tim-3 expression in NK cells in the presence of TNF-α inhibitor or NF-κB inhibitor (Fig. 4e). These results clearly demonstrate that TNF-α is able to induce the expression of Tim-3 on NK cells through NF-κB signaling pathway.

As Tim-3 expression could be regulated by TNF-α as aforementioned, we then determined TNF-α level in human esophageal tumor tissues. The data from The Cancer Genome Atlas (TCGA) were used to analyze the differential expression of TNF-α and Tim-3 in tumor tissues and adjacent tissues from esophageal cancer patients. We found that both TNF-α and Tim-3 were highly expressed in tumor tissues compared to adjacent tissues. More importantly, there is a significant correlation between TNF-α and Tim-3 expression in esophageal tumor tissues (Additional file 1: Figure S2a). These results were further verified by analyzing a cohort of 56 patients with esophageal cancer in this study (Additional file 1: Figure S2b).

### NK cell subsets are altered in a murine model with carcinogen-induced esophageal cancer

**Fig. 5:**
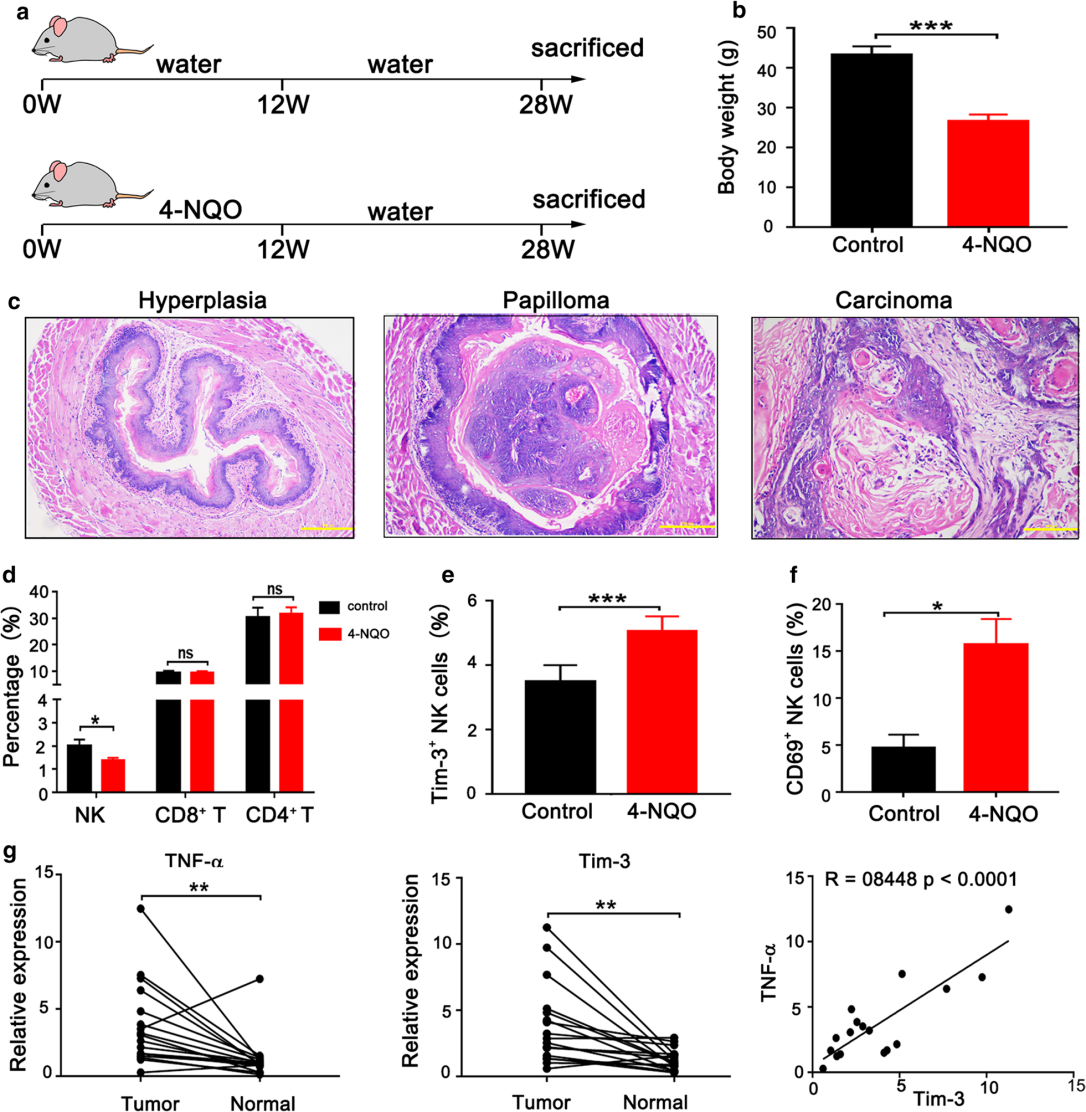
Characterization of NK cells in a murine esophageal cancer model. **a** Scheme of 4-NQO-induced carcinogenesis in C57BL/6 mice. **b** Average body weight of mice in control group and 4-NQO group before sacrifice. **c** Histological analysis of 4NQO-induced esophageal lesions in mice (×200). **d** Phenotypic analysis of splenocytes isolated from control group (n = 8) or 4-NQO group (n = 17) using flow cytometry. Flow cytometric analysis of Tim-3^+^ NK cells (**e**) and CD69^+^ NK cells (**f**) in splenocytes from control group (n = 5) or 4-NQO group (n = 10). **g** Relative mRNA levels of TNF-α and Tim-3 in esophageal tumor tissues and adjacent normal tissues from 4-NQO-induced mice (left panel) and a strong correlation between TNF-α and Tim-3 expression in esophageal tumor tissues (right panel). *p < 0.05; **p < 0.01; ***p < 0.001; ns, not significant

Our clinical data suggested that the lower frequency of NK cells is correlated to Tim-3 overexpression on NK cells in esophageal cancer patients. To further verify this relationship, we established a 4-NQO-induced esophageal tumor model in C57BL/6 mice. Esophageal cancer was induced in mice by using the drinking water containing 100 μg/mL 4-NQO for 12 weeks followed by a 16-week waiting period (Fig. 5a). Weight loss is a noticeable sign of esophageal cancer. We observed a significant reduction of average body weight in animals exposed to 4-NQO (Fig. 5b). Histological examination of tissue sections from 4-NQO-treated mice revealed pathological evidence of esophageal carcinogenesis, from hyperplasia to carcinoma (Fig. 5c). Next, we characterized NK cells in spleens from two groups of mice using flow cytometry. The percentage of NK cells statistically decreased in 4-NQO-treated mice, but there was no significant difference of CD8^+^ T cells or CD4^+^ T cells (Fig. 5d). Meanwhile, we found that the frequency of Tim-3^+^ NK cells from 4NQO-treated mice was also higher than that from control mice (Fig. 5e). Moreover, NK cells in 4NQO-treated mice expressed higher levels of the activation marker CD69 (Fig. 5f). To explore the potential contribution of TNF-α to Tim-3 expression, we examined mRNA levels of TNF-α and Tim-3 in esophageal lesions and adjacent tissues from 4-NQO-treated mice using qPCR. Both TNF-α and Tim-3 were profoundly higher in esophageal lesions compared with adjacent tissues, and, more importantly, there was a strong correlation between TNF-α and Tim-3 expression in esophageal lesions (Fig. 5g). These results suggest significant alterations of NK cell subsets in 4-NQO-induced animal model of esophageal carcinoma.

### The frequency of Tim-3^+^ NK cells correlates with tumor progression in patients with esophageal cancer

**Fig. 6:**
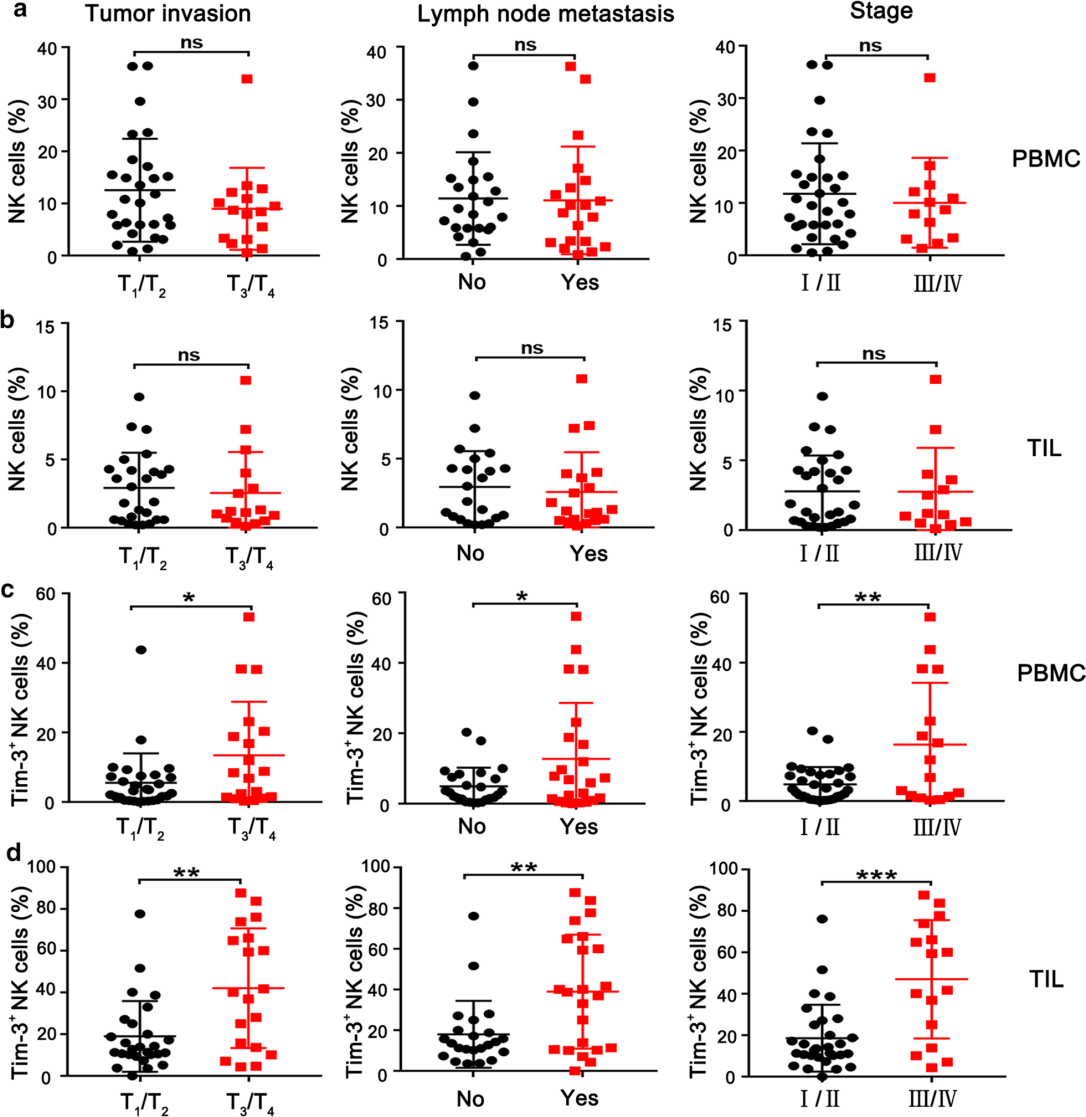
Tim-3^+^ NK cells are significantly associated with pathological parameters for poor prognosis in patients with primary esophageal cancer. The frequency of NK cells according to tumor invasion, lymph node metastasis and pathological stage in PBMC (**a**) and TIL (**b**), respectively. The frequency of Tim-3^+^ NK cells according to tumor invasion, lymph node metastasis and pathological stage in PBMC (**c**) and TIL (**d**), respectively. *p < 0.05; **p < 0.01; ***p < 0.001; ns, not significant

To discover the possible clinical significance, we analyzed the relationship between total NK cells and key pathological parameters in 46 patients with esophageal cancer, according to the UICC/AJCC Cancer Staging System, 8th Edition. There was no significant association between the frequency of peripheral NK cells and tumor invasion, lymph node metastasis and clinical stage (Fig. 6a). Similarly, no significant correlation was found between tumor-infiltrating NK cells and these pathological parameters (Fig. 6b). However, the frequency of peripheral Tim-3^+^ NK cells correlated with tumor invasion, lymph node metastasis and clinical stage (Fig. 6c). Furthermore, we observed a more significant correlation between tumor-infiltrating Tim-3^+^ NK cells and the three pathological parameters (Fig. 6d). In general, tumor invasion, lymph node metastasis and clinical stage are closely correlated with the progression of cancer. These data suggest that Tim-3^+^ NK cells may be a potential prognostic for esophageal cancer patients.

## Discussion

NK cells and CD8^+^ T cells play a crucial role in eradicating cancer cells, and inhibition of their functions is a key mechanism of tumor immune escape. T cell exhaustion has been extensively studied in different types of cancers, while the dysfunction of NK cells remains largely unknown. In this study, we detected Tim-3^+^ NK cells in esophageal cancer patients and tumor-bearing mice, and aimed to explore Tim-3-mediated NK cell dysfunction and the potential relevance with clinical prognosis.

Typically, NK cells sense the loss of major histocompatibility complex (MHC) class I molecules as a way to discriminate harmful cells. However, the induction of NK cell dysfunction by MHC class I-deficient tumor cells leads to tumor evasion from immune surveillances [5]. Functional reversal of exhausted NK cells appears to be a great potential for the treatment of cancer [9, 29]. PD-1 was reported as a marker of exhausted NK cells in digestive cancer patients, and blocking PD-1/PD-L1 signaling can restore the functions of NK cells [10]. Recent studies have reported Tim-3 is an exhaustion maker for NK cells and blocking Tim-3 signaling can increase the antitumor activity of NK cells in lung adenocarcinoma and melanoma [19, 20]. However, the role of Tim-3 in NK cells has not yet been well investigated in esophageal cancer. Here, we observed a significant increase of Tim-3 expression on NK cells from esophageal cancer patients in comparison with healthy donors. Notably, tumor-infiltrating Tim-3^+^ NK cells exhibited signs of dysfunction. It has been reported that co-expression of PD-1 and Tim-3 was associated with functionally exhausted NK cells in colon cancer tissues [5]. However, co-expression of inhibitory immune checkpoints was not predominantly observed among tumor-infiltrating NK cells in this study. Further studies are needed to define the function of NK cell subsets expressing different immune checkpoints.

Conventionally, NK cells are divided into two major subsets: CD56^bright^ and CD56^dim^ NK cells. Watanabe et al. reported the down-regulated CD16 and up-regulated CD56 molecules on NK cells of esophageal cancer patients, resulting in NK cell dysfunction [30]. A prevalence of CD56^bright^ NK cells has also been observed in other solid malignancies, such as an increase of CD56^bright^perforin^low^ NK cells in human breast and lung cancers [31, 32]. Recently, it was reported that CD56^bright^ NK cells had markedly higher PD-1 expression relative to CD56^dim^ NK cells in Hodgkin lymphoma patients, indicating an exhausted NK cell subset to induce immune evasion [33]. However, Pesce et al. showed that PD-1 expression is restricted to CD56^dim^ NK cell subset, representing terminally differentiated NK cells with impaired functions [34]. To date, little is known regarding Tim-3 expression on NK cell subsets. Here, we observed that Tim-3 expression is more significantly up-regulated on CD56^bright^ NK cells from esophageal cancer patients, especially in tumor microenvironment. However, whether Tim-3 expression on different NK cell subsets has different functions needs to be further addressed.

We attempted to explore the mechanisms involved in Tim-3 expression of NK cells in esophageal cancer microenvironment. Ndhlovu et al. reported that peripheral NK cells expressed high levels of Tim-3 after stimulation with IL-2, IL-12, IL-15 or IL-18 in vitro [35]. In this study, we identified for the first time that TNF-α could induce significant Tim-3 expression on NK cells from healthy donors in vitro. Whether this reflects the responsiveness of tumor-infiltrating NK cells remains to be determined. TNF-α is a multifunctional cytokine produced by activated immune cells, tumor cells or stromal cells in tumor microenvironment [36–38]. The NF-κB cascade can be activated by inflammatory cytokines such as TNF-α. Our results showed that NF-κB inhibitor blocked Tim-3 expression on NK cells induced by TNF-α, suggesting that NF-κB mediates the expression of Tim-3. The remarkable association between Tim-3 and TNF-α in both human and murine esophageal tumor tissues further support the important role of TNF-α in Tim-3 expression on NK cells.

Accumulating evidence indicates the low density of NK cells in different forms of cancers such as melanoma, lung cancer, breast cancer, renal cell cancer and gastrointestinal tumors [39–42]. The activating receptors NKp30 and NKp46 have been identified as predictive markers for a good prognosis in acute myeloid leukemia and metastatic prostate cancer [43, 44]. However, the prognostic value of co-inhibitory receptors on NK cells has been very barely studied, especially in esophageal cancer. Our analyses of human cancer samples clearly indicate that Tim-3^+^ NK cells are significantly associated with pathological parameters for poor prognosis of esophageal cancer. This implies that the dysfunction of NK cells may contribute to the progression of the disease in esophageal cancer patients. It would be of great interest to establish whether Tim-3^+^ NK cells can serve as a predictive biomarker for the outcome of esophageal cancer patients.

## Conclusions

The data presented in this study suggest that Tim-3 may act as a novel marker for NK cell dysfunction associated with poor prognostic factors in esophageal cancer patients. Our findings highlight the potential of Tim-3 as a promising therapeutic target to restore the functions of NK cells.

## Additional file

**Additional file 1.** Additional figures and tables.

## Data Availability

Authors can confirm that all relevant data and materials are available on request from the authors.

## Abbreviations

MHC: major histocompatibility complex
NK: natural killer
PBMC: peripheral blood mononuclear cells
PD-1: programmed cell death protein 1
TCGA: The Cancer Genome Atlas
TIL: tumor infiltrating lymphocytes
Tim-3: T-cell immunoglobulin domain and mucin domain-3
TNF-α: tumor necrosis factor-α
