# Supplementary material for: TNF-α-induced Tim-3 expression marks the dysfunction of infiltrating natural killer cells in human esophageal cancer
Source: J Transl Med. 2019 May 20;17:165. doi: 10.1186/s12967-019-1917-0 (PMC6528366; doi:10.1186/s12967-019-1917-0)
Supplement: Supplementary file 1 — Additional file 1. Additional figures and tables. [file 12967_2019_1917_MOESM1_ESM.docx]

***Journal of translational medicines* (submitted in 2019) – Yujia Zheng et al.**

**
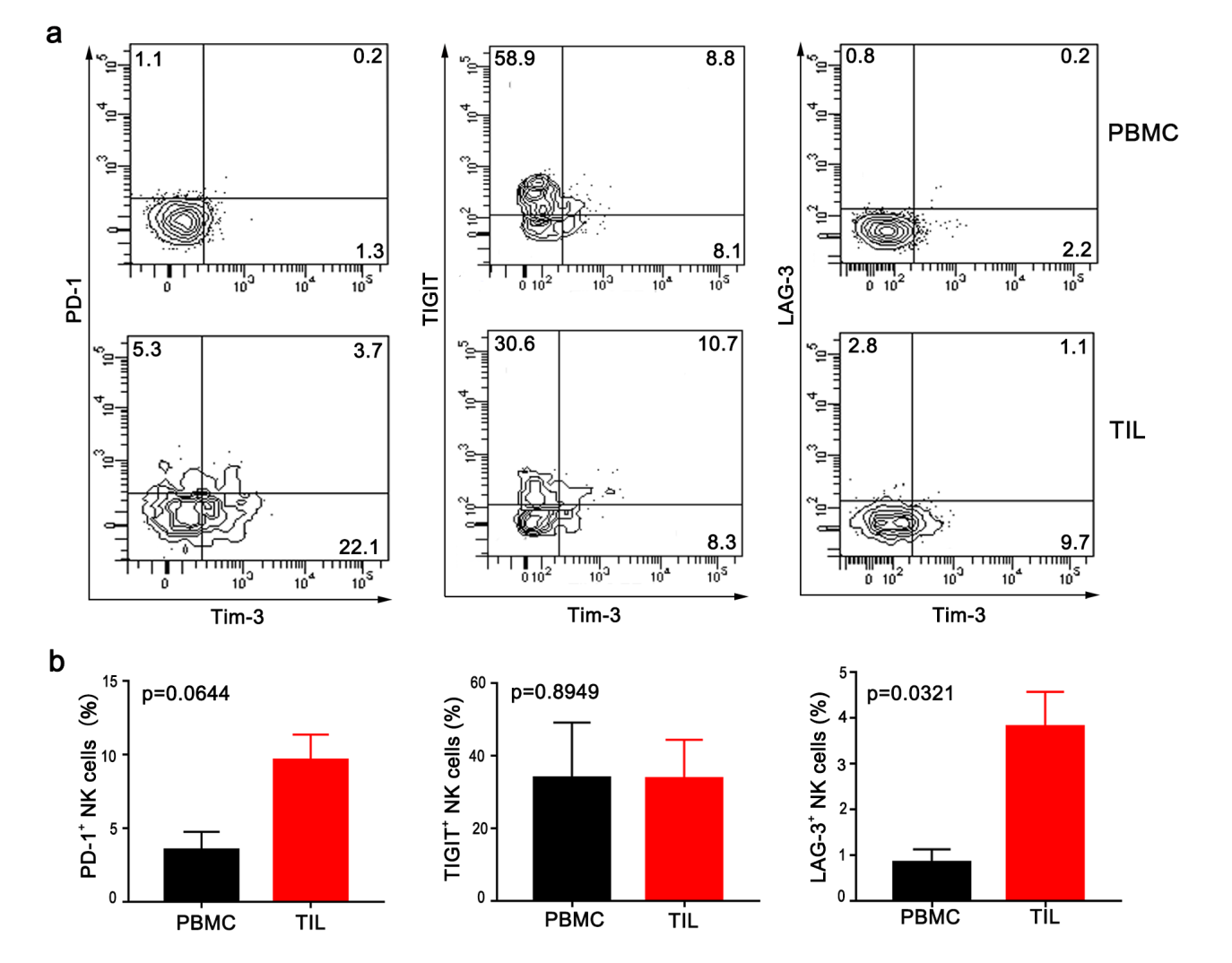
**

**Additional Figure S1** Expression of co-inhibitory molecules on circulating or intratumoral NK cells from 5 esophageal cancer patients, shown by representative flow cytometric plots (**a**) and statistical analysis (**b**).


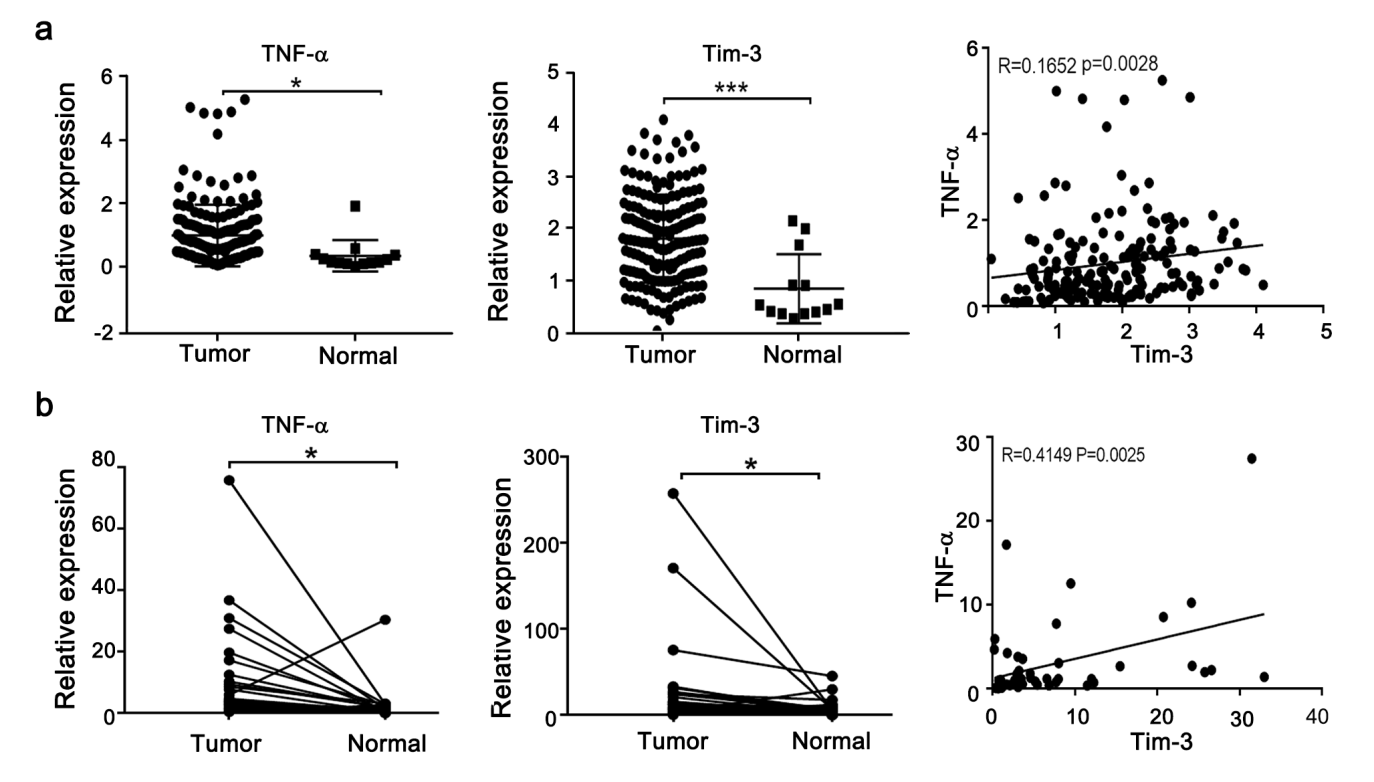


**Additional Figure S2** Strong correlation between TNF-α and Tim-3 mRNA levels in human esophageal cancer tissues. Relative mRNA levels of TNF-α (left panel) and Tim-3 (middle panel), and their expression relationship (right panel) in tumor tissues from esophageal cancer patients using TCGA dataset (**a**) or a cohort in this study (**b**). **p*<0.05; ****p*<0.001.

**Additional Table S1** Clinicopathological features of 52 patients with esophageal cancer

|  | **Clinical feature** | **N=52** |  |
| --- | --- | --- | --- |
|  | **Gender**  Male Female  **Age**（**years**）  <60  ≥60  **Tumor infiltration**  Lamina propria or submucosa Muscularis propria Adventitia  Adjacent structures  **Lymph node metastasis**  Present Absent  **Pathological stage**  I, II III, IV | 19  33  17  35  12  20  18  2  24  28  36  16 |  |

**Additional Table S2** Fluorophore-conjugated antibodies

| Antibody | Fluorophore | Manufacturer |
| --- | --- | --- |
| Human anti-CD3 | APC-CY7, FITC, PE-CY7 | BioLegend |
| Human anti-CD56 | PE, PE-CY7, APC-CY7 | BioLegend |
| Human anti-Tim-3 | PE, APC | BioLegend |
| Human anti-PD-1 | APC, FITC | BioLegend |
| Human anti-LAG-3 | FITC | BioLegend |
| Human anti-TIGIT | APC | BioLegend |
| Human anti-CD69 | APC | BioLegend |
| Human anti-Annexin V | APC | BioLegend |
| Human anti-IFN-γ | APC | BioLegend |
| Human anti-CD107a | FITC | BioLegend |
| Mouse anti-CD3 | APC-CY7 | BioLegend |
| Mouse anti-CD49b | PE-CY7 | BioLegend |

**Additional Table S3** PCR primer sequences

| Gene | Forward 5′- 3′ | Reverse 5′-3′ |
| --- | --- | --- |
| Human GAPDH | GGAGCCAAAAGGGTCATCACTC | GAGGGGCCATCCACAGTCTTCT |
| Human Tim-3 | CTGCTGCTACTACTTACAAGGTC | GCAGGGCAGATAGGCATTCT |
| Human IFN-γ | TCGCCTGATTACACAGATGAA | TCGTGTGTGCTTCTCCACTC |
| Human granzyme B | CCCCTGGGAAAACACTCACA | GCCTCCAGAGTCCCCCTTA |
| Human perforin | ATTCCAGAGCCCAAGTGCC | CCTTTGTGTGTCCACTGGGA |
| Human NKG2D | GAGTGATTTTTCAACACGATGGC | ACAGTAACTTTCGGTCAAGGGAA |
| Human TNF-α | CCTCTCTCTAATCAGCCCTCTG | GAGGACCTGGGAGTAGATGAG |
| Murine GAPDH | TGACCTCAACTACATGGTCTACA | CTTCCCATTCTCGGCCTTG |
| Murine TNF-α | CAGGCGGTGCCTATGTCTC | CGATCACCCCGAAGTTCAGTAG |
| Murine Tim-3 | TCAGGTCTTACCCTCAACTGTG | GGCATTCTTACCAACCTCAAACA |
